# The present status of metformin in fertility-preserving treatment in atypical endometrial hyperplasia and endometrioid endometrial cancer

**DOI:** 10.3389/fendo.2022.1041535

**Published:** 2022-10-27

**Authors:** Jun Guan, Xiao-Jun Chen

**Affiliations:** ^1^ Department of Gynecology, Obstetrics and Gynecology Hospital, Fudan University, Shanghai, China; ^2^ Shanghai Key Laboratory of Female Reproductive Endocrine Related Diseases, Shanghai, China

**Keywords:** metformin, fertility-preserving treatment, progestin, endometrial cancer, atypical endometrial hyperplasia, review

## Abstract

Progestin therapy is the main fertility-sparing treatment for women with endometrial cancer (EC) and atypical endometrial hyperplasia (AEH). However, still 15-25% of these women failed to achieve complete response (CR) and then lost their fertility after definitive surgery. Metformin has been demonstrated to play an anti-cancer role in multiple cancers including EC. Several studies also suggested metformin had potential benefit in improving the therapeutic outcome of fertility-preserving treatment alongside with progestin. This review has discussed existed evidence regarding the effect of metformin combined with progestin for women with AEH and EC who desire childbearing. Nevertheless, the therapeutic effect of metformin varied in different studies due to the high heterogeneity in the patient’s characteristics, the inconsistency in dose and treatment duration of metformin, the combined use of hysteroscopy, the insufficient sample size and underpowered study-design. Therefore, care should be taken when interpreting the current results on this issue. Till now, there is still no strong evidence supporting the use of metformin in fertility-preserving treatment in AEH and EEC patients. Further research is needed to provide high-quality data to validate the role of metformin as adjunctive therapy alongside with progestin to preserve fertility for AEH and EEC patients.

## Introduction

Endometrial cancer has become the most common gynecologic cancer in high-income countries and areas (1). The incidence is rising, with an increasing number of women diagnosed in their reproductive age (1). Progestin is the main regimen of fertility-preserving treatment for young women diagnosed with well-differentiated endometrioid endometrial cancer (EEC) and atypical endometrial hyperplasia (AEH). However, the remission rates of progestin were 75%-85% for EC and AEH (2, 3). Hence, 15-25% of the patients failing to achieve CR had to receive definitive surgery and lost their fertility permanently. Prolonged progestin treatment brings more side effects such as weight gain, thromboembolism, hypertension, etc (4). In this context, seeking better fertility-sparing regimen to achieve higher CR rate with less side effect has become an important issue.

Previous studies revealed that women with obesity, insulin resistance, or diabetes might be at higher risk of EEC (3). This has drawn an amount of attention on whether medication targeted on glucose metabolism might improve the therapeutic outcomes of EEC, particularly as potential adjunctive therapy for patients who desired to preserve fertility (5). Metformin, therefore, has aroused great interest to be used for EEC patients. As a biguanide antidiabetic agent, metformin usually has been used to treat type II diabetes mellitus with good tolerance and low toxicity (6). Preliminary studies suggest that metformin may be a beneficial adjunctive therapy with a synergistic effect alongside progestin treatment in AEH or EEC (7–9). However, evidence from clinical studies varied. This paper reviewed current findings regarding the role of metformin in fertility-sparing treatment for AEH and EEC patients.

## Materials and methods

All searches were conducted from 1^st^ January 2022 to 1^st^ February 2022 for this narrative review, using key words and subject headings in databases of Cochrane, PubMed MEDLINE, Web of Science, CINAHL, LILACS, and clinicaltrials.gov. Manual searches were also conducted for reference lists of each selected articles and other relevant studies that could have been missed during database searches.

### The mechanism of metformin inhibiting tumor cells in basic studies

Experimental studies have demonstrated that metformin may suppress the growth of breast, ovarian, prostate, and EC cells *via* altering glucose metabolism and inhibiting the phosphatidylinositol-3-kinase/protein kinase B/mammalian target of rapamycin (PI3K-AKT-mTOR) signaling pathway (5, 10). Also, metformin has been found to alternate the adenosine monophosphate/adenosine diphosphate/adenosine triphosphate (AMP/ADP/ATP) ratio, to activate adenosine monophosphate-activated protein kinase (AMPK), a major mediators of cell energy homeostasis, then to induce tumor suppressor genes to inhibit the proliferation of EC cells (11). Notably, a recent finding showed low-dose metformin could activate AMPK without effects on cellular AMP levels. The study found that metformin activates AMPK through binding to Presenilin enhancer 2 (PEN2) to inhibit the lysosomal proton pump v-ATPase (12). In addition, metformin has been shown to increase expression of the progesterone receptor and sensitize progestin-resistant EC cells to medroxyprogesterone (MPA)-induced apoptosis (13, 14).

### The correlation between metformin usage and endometrial cancer prognosis in clinical studies

Benefit of metformin has also been reported in multiple clinical studies in terms of improving the overall survival and decrease the recurrence rate for EC patients (10). A meta-analysis reviewed 19 eligible clinical research and concluded that metformin might be associated with reversion of AEH to a normal endometrial (10). The study also found that among EC patients, metformin-users had a higher overall survival compared with metformin non-users and non-diabetic patients [Hazard Ratio (HR) 0.82, 95% Confidence Interval (CI) 0.70–0.95, p = 0.09), despite the high heterogeneity of the included studies (10). Several non-controlled clinical trials showed significantly decreased proliferation biomarkers staining after metformin treatment, such as nuclear antigen Ki 67(Ki67), phosphor-protein ribosomal S6 (pS6), phosphor- 4E- binding protein 1 (p4E – BP1), and phosphomonophosphate adenosine kinase(pAMPK) (10). Also, a retrospective cohort study including 349 stage III-IV or recurrent EC patients showed a significant increase in overall survival (HR 0.40, p = 0.0036) in the metformin-treated group compared with the non-metformin group during chemotherapy (15). Furthermore, a large-scale cohort study (n=985) reported that diabetic women with non-endometrioid EC who received metformin had a significant better overall survival rate (HR 0.54, 95% CI 0.30–0.97, p = 0.04) than diabetic EC patients without taking metformin (16). Additionally, in obese women with EEC, the recurrence rates were 1.9% (n = 1/64) in women using metformin compared with 10.3% (n=25/287) in those without metformin use (p=0.05) (17).

### Metformin in fertility-preserving treatment

Since the anti-tumor effect of metformin has been found in basic and clinical studies, it seems reasonable to add metformin into fertility-sparing treatment to improve the therapeutic response for AEH and EEC patients. However, data from clinical studies failed to provide strong evidence supporting the usage of metformin in fertility preserving treatment (7, 18). Till now, the effect of metformin combined with progestin as fertility-sparing treatment has been majorly investigated in six clinical studies, including two randomized controlled trials, one perspective non-controlled trial, one perspective pilot study, and two retrospective studies. The findings of these studies are summarized and discussed below.

### The effect of metformin on complete response of fertility-preserving treatment

Currently, it seems no consistency has been achieved yet regarding the benefit of adding metformin into progestin therapy to preserve the fertility for AEH/EEC women. Metformin has been reported to improve the remission rate of progestin in AEH and EC patients in several studies (7–9, 19, 20). Shan et al. firstly reported a CR rate of 75% (6/8) of AEH women treated with metformin plus megestrol acetate (MA), compared with 25% (2/8) in those received MA alone in a prospective pilot study (9). Based on these findings, a randomized controlled trial (RCT) was further conducted and then presented the potential advantage of metformin in improving the early CR rate of oral progestin (7). This phase-II clinical trial enrolled 150 (123 AEH, 27 EEC) patients with median body mass index (BMI) of 24.7kg/m^2^ showed a borderline-significantly higher CR rate within 16 weeks of treatment in the metformin (1500mg daily) plus MA (160mg daily) group compared with the MA-only group [34.3% vs 20.7%, Odds Ratio (OR) 2.0, 95% CI 0.89–4.51, p = 0.09]. In subgroup analysis, this difference in 16-week CR rate became statistically significant in 102 AEH patients (39.6% vs 20.4%, OR 2.56, 95% CI 1.06–6.21, p = 0.04), also in non-obese (51.4 vs 24.3%, OR 3.28, 95% CI 1.22–8.84, p = 0.02) and insulin sensitive (54.8 vs 28.6%, OR 3.04, 95% CI 1.03–8.97, p = 0.04) subgroups of AEH women. However, the difference in CR rate became smaller at 32 weeks of treatment between groups of metformin plus MA and MA alone (74.3% vs 68.2%, p=0.43). Furthermore, similar trend was reported in a perspective single-arm study of 36 AEH/EEC patients with mean BMI of 31 kg/m^2^ who received medroxyprogesterone acetate (MPA, 400 mg/day) and metformin (750–2250 mg/day) for 24–36 weeks to preserve the fertility (8). The 36-week CR rates of metformin plus MPA was 81%, which was higher than that (64%) of patients receiving MPA alone in previously most comparable study. Besides, a retrospective study showed that the 6-month CR rate was significantly improved in women received metformin combined with levonorgestrel-intrauterine device (LNG-IUD) compared with LNG-IUD alone (86.7% vs 58.9%, HR 2.31, 95% CI 1.09-4.89, p=0.030), with a median BMI of 39.9 kg/m^2^ (19).

Conversely, several studies showed the similar therapeutic effect of progestins with or without metformin as fertility-sparing treatment. A multi-center study retrospectively analyzed 92 cases (38 EC and 54 AEH) with median BMI of 37.7 kg/m^2^ who received progestins (MA, MPA, or LNG-IUD) (21). Of them, 34 women also received metformin for > 3 months prior the progestin use or within 3 months after initiating progestin. The difference in median duration of treatment to CR was not significant between metformin recipients and non-recipients (6.0 vs 4.9 months, p=0.31), after adjusting diabetic status and BMI. No difference was found in overall CR rate. Another retrospective study presented that in AEH patients receiving oral progestin, the cumulative 6-month CR rates were similar in women treated with or without metformin (23.1% vs 27.8%, p=0.384) after adjusting diabetes status (19). A recent phase II randomized controlled trial showed similar 6-month CR rates in women using LNG-IUD alone or LNG-IUD plus metformin [61% (20/33, 95%CI 42–77%) vs. 57% (24/42, 95% CI 41–72%)] (22). This trial enrolled 165 obese women (BMI of 48 kg/m^2^, 69 AEH and 96 EEC) who were willing to maintain fertility or intolerant to surgery. The mean age was 53 years old, and a half of participants was postmenopausal. Nevertheless, no subgroup analysis was performed for young patients desiring fertility-sparing therapy in this trial. A recent meta-analysis found that the OR for remission was not statistically different between metformin plus progestin and progestin-alone therapies (pooled OR 1.35, 95%CI 0.91–2.00, p=0.14) (18).

Collectively, there might be several reasons for the inconsistency of these research findings.

Firstly, there was heterogeneity in patient’s characteristics, administration of progestins and metformin among all studies. The BMI of recruited women were ranged from 24.7 to 48kg/m^2^, with many missing data that impacted statistical comparison between metformin plus progestin and progestin alone (7–9, 18–22). One study analyzed total CR rates for all participants including pre- and postmenopausal women without stratification on age (22). The progestin protocols, also, were various among studies including MPA, MA, and LNG-IUD, and the doses of metformin ranged from 500 mg daily to 2250 mg daily or was unspecified, and the duration of metformin therapy was also ranged from at least 6 months to nearly 18 months (7–9, 18–22).

Secondly, most studies reported outcomes for AEH and EEC together, without separating patients with AEH or EEC for independent analyses. Most patients were with AEH, which might bring the bias because the response rate of AEH was usually better than that of EEC. A randomized controlled trial (n=150) supported the merit of metformin in improving the early CR rate at 16 weeks of treatment for AEH patients, even for AEH women without obesity, insulin resistance, hypertension, or diabetes. These results from subgroup analyses are promising, but not yet strong enough to be included in clinical routines. Phase III trials are needed to further validate the effect of metformin (7).

Thirdly, the long-term benefit of metformin might also be concealed by repeated hysteroscopy or curettage every 3-6 months during the progestin therapy. Hysteroscopy has been recently reported to improve the response rate to progestin due to its advantage in complete removal of endometrial lesions (23, 24). A large-scale (n= 152) study reported an improved 12-month CR rate of 88.9% in AEH and 91.4% in EEC patients by hysteroscopy combined with MA (23). A systematic review of 39 years of published studies of young early EC patients reported a CR rate of 88.9% after being treated with hormone therapy combined with hysteroscopy for fertility preservation (25). Falcone et al. also reported a complete regression rate of 96.3% with a recurrence rate of 7.7% in early-stage EC patients who underwent hysteroscopic resection and progestin therapy (26). Laurelli et al. described that 78% of the patients with early EC achieved CR after receiving hysteroscopy plus LNG-IUD, and only 7% of patients had a cancer recurrence (24). This might help to explain why in some studies the total CR rate during a median treatment time of 6-12 months were similar in patients receiving progestin with or without metformin.

Lastly but importantly, current studies were mostly retrospective studies, prospective single-arm, or pilot studies with small sample size. Although one phase II RCT recruiting 150 patients with AEH or EEC showed no significant difference in CR rate between groups of metformin plus MA and MA alone (7), the sample size was not sufficient to provide strong evidence supporting or against the usage of metformin. A larger adequately powered RCT is still required to better reveal the role of metformin in progestin-based therapy for AEH and EEC patients.

### The effect of metformin on relapse rate

Metformin showed the advantage in reducing the relapse rate in progestin therapy. Up to our knowledge, to date, only three studies analyzed relapse rate between metformin with progestin and progestin alone in fertility-sparing treatment (7, 8, 21). Two of the studies were retrospective, and one was a randomized trial. Metformin plus progestin were found to be correlated with lower recurrence rate of AEH/EEC than progestin alone (8, 21). In a multi-center retrospective study (n=92), the relapse rates were lower in metformin users than in metformin non-users (17.4% vs. 25%, OR 0.63, 95%CI 0.17–2.3) in progestin therapy for AEH/EEC patients (21). A non-controlled perspective study analyzing metformin plus MPA also showed a relapse rate of 10% which were much lower than 47% in previously most comparable study (8). However, a phase II RCT showed metformin plus MA and MA-alone groups shared similar relapse rates (10.1% vs 9.1%, OR1.01, 95% CI 0.31–3.26) (7). In the trial, the repeated hysteroscopy used every three months might also help to remove the disease, which might result in the similar relapse rate between metformin plus MA and MA alone group. A recent meta-analysis included all three existed studies and reported a significantly lower relapse rate in metformin plus progestin group compared with progestin-alone group (pooled OR 0.46, 95% CI 0.24–0.91, p=0.03). Results from subgroup analysis on only retrospective studies also showed the similar trend (OR 0.30, 95% CI 0.13–0.72, p<0.01) (18).

### The effect of metformin on fertility outcome

No significant effect of metformin has been found regarding reproductive outcomes based on three existed studies. In a phase-II RCT, after almost 2-year follow up, the pregnancy rates were similar between patient receiving metformin plus MA and MA alone (48.4% vs. 41.9%, OR 1.13, 95% CI 0.43–2.93) (7). The different utilization of assisted reproductive technology, the insufficient metformin usage and more obese and diabetic patients in metformin-treated cohorts may contribute to this conclusion. Nevertheless, further research with sufficient data is needed to provide this information.

### Adverse events of metformin in fertility-sparing treatment

The incidence of adverse events was showed in two studies (7, 22). In one study, no significant difference in adverse events was found between the treatment arms (22). In another study, weight gain was presented as the most common side effect during the treatment, occurring in 34.2% of women treated by metformin plus MA compared with 41.9% in women receiving MA only (7). During the treatment, median weight gain in the metformin plus MA group was 2.5 kg compared with 5.0 kg in the MA-only group (p = 0.01) (7). Except for grade 1-2 diarrhea, other adverse events appeared less likely to occur in the patients who received metformin plus MA group than those received MA alone (7). Metformin seemed to be safe and well tolerant in fertility sparing therapy and may also help to reduce the weight gain caused by oral progestin.

In conclusions, metformin seems to be promising in improving the early complete response rate and reducing the relapse rate of progestin therapy in AEH and EEC patients, with no significant effect on reproductive outcomes based on existed studies. Metformin seemed to be safe and well tolerant in fertility sparing therapy and may also help to reduce the weight gain caused by oral progestin. However, no consistency has been reached yet regarding the benefit of combining metformin and progestin in AEH and EEC patients. The therapeutic effect of metformin varied in previous studies because of the high heterogeneity in the patient’s characteristics, the inconsistency in dose and treatment duration of metformin, the combined use of hysteroscopy, the insufficient sample size, the limited number of studies and underpowered study-design. Thus, care should be taken to interpret the results and there was still no robust evidence supporting the use of metformin in fertility-preserving treatment in AEH and EEC patients. An ongoing phase III trial (FELICIA trial) investigating the appropriate doses of metformin plus MPA for AEH and EEC patients to maintain the fertility will provide further data in the future (registry number: jRCT2031190065) (27). More high-quality clinical trials are needed to confirm the value of metformin in fertility-preserving treatment for AEH and EEC patients.

## Author contributions

JG: Conceptualization, Methodology, Investigation, Writing - Original Draft; X-JC: Supervision; Conceptualization, Resources, Supervision, Writing - Review and Editing. All authors contributed to the article and approved the submitted version.

## Conflict of interest

The authors declare that the research was conducted in the absence of any commercial or financial relationships that could be construed as a potential conflict of interest.

## Publisher’s note

All claims expressed in this article are solely those of the authors and do not necessarily represent those of their affiliated organizations, or those of the publisher, the editors and the reviewers. Any product that may be evaluated in this article, or claim that may be made by its manufacturer, is not guaranteed or endorsed by the publisher.

## Abbreviations

EC, endometrial cancer; EEC, endometrioid endometrial cancer; AEH; atypical endometrial hyperplasia; CR, complete response; MA, megestrol acetate; MPA, medroxyprogesterone; RCT, randomized controlled trial; BMI, body mass index; CI, confident interval; OR, odds ratio; HR, hazard ratio; Ki-67, nuclear antigen Ki 67; pAMPK, phosphomonophosphate adenosine kinase; pS6, phosphor-protein ribosomal S6; PEN2, presenilin enhancer 2; P4E – BP1, phosphor- 4E- binding protein 1; PI3K-AKT-mTOR, phosphatidylinositol-3-kinase/protein kinase B/mammalian target of rapamycin; AMP/ADP/ATP, adenosine monophosphate/adenosine diphosphate/adenosine triphosphate.
